# External and Genetic Conditions Determining Male Infertility

**DOI:** 10.3390/ijms21155274

**Published:** 2020-07-24

**Authors:** Piotr Kamiński, Jędrzej Baszyński, Izabela Jerzak, Brendan P. Kavanagh, Ewa Nowacka-Chiari, Mateusz Polanin, Marek Szymański, Alina Woźniak, Wojciech Kozera

**Affiliations:** 1 Department of Biotechnology, Institute of Biological Sciences, University of Zielona Góra, Prof. Szafran St. 1, PL 65-516 Zielona Góra, Poland; 2 Department of Ecology and Environmental Protection, Department of Medical Biology and Biochemistry, Collegium Medicum in Bydgoszcz, Faculty of Medicine, Nicolaus Copernicus University in Toruń, M. Skłodowska-Curie St. 9, PL 85-094 Bydgoszcz, Poland; jedrzej.baszynski@cm.umk.pl; 3 Department of Pharmacology and Toxicology, Collegium Medicum, University of Zielona Góra, Zyta St. 28, PL 65-046 Zielona Góra, Poland; izabelajerzak@wp.pl; 4 RCSI Biology, Royal College of Surgeons in Ireland, 123 St. Stephen’s Green, Dublin 2, Ireland; bkavanagh@rcsi.ie; 5 Department of Sport Promotion, Institute of Biological Sciences, University of Zielona Góra, Prof. Szafran St. 1, PL 65-516 Zielona Góra, Poland; e.chiari@wnb.uz.zgora.pl; 6 Karol Marcinkowski University Hospital in Zielona Góra, Zyta St. 26, PL 65-045 Zielona Góra, Poland; mateusz.polanin@gmail.com; 7 Female Pathology and Oncological Gynecology, Department of Obstetrics, Faculty of Medicine, University Hospital No. 2, Collegium Medicum in Bydgoszcz, Nicolaus Copernicus University in Toruń, Ujejski St. 75, PL 85-168 Bydgoszcz, Poland; kikpoloz@cm.umk.pl; 8 NZOZ Medical Center Co. Prof. dr. hab. med. Wiesław Szymański, Dr. hab. med. Marek Szymański, Waleniowa St. 24, PL 85-435 Bydgoszcz, Poland; 9 Department of Medical Biology and Biochemistry, Faculty of Medicine, Collegium Medicum in Bydgoszcz, Nicolaus Copernicus University in Toruń, Karłowicz St. 24, PL 85-092 Bydgoszcz, Poland; alina-wozniak@wp.pl; 10 Department of Agricultural Chemistry, Faculty of Agriculture and Biotechnology, UTP University of Science and Technology in Bydgoszcz, Seminaryjna St. 5, PL 85-326 Bydgoszcz, Poland; kozera@utp.edu.pl

**Keywords:** male infertility, environmental stressors, trace elements, pro-antioxidant mechanisms, polymorphisms, AZF-microdeletions, cystic fibrosis, congenital bilateral absence of vas deferens

## Abstract

We explain environmental and genetic factors determining male genetic conditions and infertility and evaluate the significance of environmental stressors in shaping defensive responses, which is used in the diagnosis and treatment of male infertility. This is done through the impact of external and internal stressors and their instability on sperm parameters and their contribution to immunogenetic disorders and hazardous DNA mutations. As chemical compounds and physical factors play an important role in the induction of immunogenetic disorders and affect the activity of enzymatic and non-enzymatic responses, causing oxidative stress, and leading to apoptosis, they downgrade semen quality. These factors are closely connected with male reproductive potential since genetic polymorphisms and mutations in chromosomes 7, X, and Y critically impact on spermatogenesis. Microdeletions in the Azoospermic Factor AZF region directly cause defective sperm production. Among mutations in chromosome 7, impairments in the cystic fibrosis transmembrane conductance regulator *CFTR* gene are destructive for fertility in cystic fibrosis, when spermatic ducts undergo complete obstruction. This problem was not previously analyzed in such a form. Alongside karyotype abnormalities AZF microdeletions are the reason of spermatogenic failure. Amongst *AZF* genes, the deleted in azoospermia *DAZ* gene family is reported as most frequently deleted AZF. Screening of AZF microdeletions is useful in explaining idiopathic cases of male infertility as well as in genetic consulting prior to assisted reproduction. Based on the current state of research we answer the following questions: (1) How do environmental stressors lessen the quality of sperm and reduce male fertility; (2) which chemical elements induce oxidative stress and immunogenetic changes in the male reproductive system; (3) how do polymorphisms correlate with changes in reproductive potential and pro-antioxidative mechanisms as markers of pathophysiological disturbances of the male reproductive condition; (4) how do environmental stressors of immunogenetic disorders accompany male infertility and responses; and (5) what is the distribution and prevalence of environmental and genetic risk factors.

## 1. Introduction

Nowadays a large pool of substances potentially harmful for human health is incessantly present in the natural environment. Toxic metals (Cd, Pb, Hg, As, Be, V, Ni), dioxins, anti-metabolites, dyes, herbicides, fungicides, or even house dust constitute a detrimental mixture that people are exposed to practically every day [1,2,3,4]. Therefore, essential systems of the human organism are continually subjected to potential damage. Among them, the reproductive system, especially spermatogenesis, appears to be affected, too [5]. Long-term exposure to destructive factors may lead to occupational diseases, irreversible changes in the reproductive system (worsening of sperm quality, disorders in spermatogenesis), or even to infertility [6]. In this respect, toxic heavy metals and certain chemical pollutants (dichloro-diphenyl-dichloro-ethane DDT or methoxychlor) are considered as oxidative stress inducers [7]. Oxidative stress is defined as a lack of balance between per-oxidation and anti-oxidation, directly connected with overproduction of reactive oxygen species ROS [8]. It is difficult to avoid certain factors that induce oxidative stress, especially in cities due to traffic and industrial activity (smog, traffic fumes), but other sources of ROS may remain under control. Cessation of smoking, introducing a low-fat diet, or regular physical activity can be simple strategies against oxidation [9]. One of the causes of oxidative stress is the decrease of antioxidant enzymes (superoxide dismutase SOD, catalase CAT or glutathione peroxidase GPx) which erodes the line of defense against reactive forms of oxygen [10]. Thus, introducing an anti-oxidative diet consisting, e.g., of fruits and vegetables rich in vitamins A, C, E, and B, is recommended and beneficial for strengthening the anti-oxidative potential of the body [11,12,13]. The male reproductive condition can be improved by supplementation of beneficial elements such as zinc or selenium that cause positive changes in sperm count and motility [14]. Melatonin, beta-carotene, or luteine also contribute to maintaining high semen quality [15,16].

Since oxidative stress contributes to serious impairments in genetic composition, such as damage of chromosomes or breakages in the deoxyribonucleic acid DNA [8], it is valuable to analyze genetic reasons for male infertility. On chromosome Y, microdeletions in the AZF-region (called the azoospermic factor) result in spermatogenic failure and a lack of sperm cells in semen [17,18]. The world frequency of AZF microdeletions is estimated in the range of 1–15% of cases of azoospermic infertile men [19,20]. Other common reason for male infertility is cystic fibrosis, i.e., a recessive disease with a frequency of occurrence of 1/2500 live births, is caused by mutations in the *CFTR* gene on chromosome 7 [21]. Overproduction of thick, sticky mucus in organs with mucous glands is a typical symptom of the disease. In addition to pathological changes in the alimentary or respiratory systems, cystic fibrosis also contributes to infertility through clogging spermatic ducts with mucus [22,23]. The condition often accompanying cystic fibrosis is a congenital bilateral absence of the vas deferens, manifested as aplasia of spermatic ducts and an obstruction of sperm outflow into the urethra. Similarly to cystic fibrosis, congenital bilateral absence of the vas deferens is caused by mutations in the *CFTR* gene [24,25]. Finally, impairments on the X chromosome play an essential role in pathogenesis of Klinefelter syndrome KS (the presence of an extra X chromosome in the male karyotype) and Kallmann KAL syndrome (mutations in the KAL1 gene on the X chromosome; KAL1 is a human gene which is located on the X chromosome at Xp22.3 and is affected in some male individuals with Kallmann syndrome). The former is manifested by small testicles, degenerative changes in spermatic ducts, azoospermia, and decay of potency [26,27,28,29,30], while the latter is manifested in a deficiency in the sense of smell, delayed maturation, small testicles, and underdevelopment of the penis [31,32,33,34].

We reviewed the recent data in an effort (1) to estimate the diversification of potentially harmful factors accumulated in the modern environment (from heavy metals to domestic dust) and their influence on human fertility; (2) to establish the relationship between various pollutants and oxidative stress intensification; (3) to find effective strategies in overcoming oxidative stress in everyday human life, thereby improving reproductive conditions; (4) to analyze common genetic factors underlying male infertility associated with chromosome Y (AZF region); and (5) to analyze the most common factors underlying male infertility associated with chromosome 7 and the X chromosome.

This review of existing research will broaden our knowledge of the impact of environmental stressors on antioxidant reactions, and changes of lipoperoxidation and immunogenetic disorders in patients with symptoms of infertility. The results can be used in the prophylaxis of male infertility among patients inhabiting degraded areas. It will also answer some questions about the causes of infertility in men in whom it was previously unknown. Linking the biochemical and morphological parameters of semen with immunogenetic disorders will bring clarification to the role of environmental factors in shaping responses to various stressors. Analysis of the activity of enzymatic antioxidative mechanisms, lipoperoxidation intensity, and the levels of stress proteins and non-enzymatic mechanisms jointly can give a more complete picture of conditions shaping the response of an organism to environmentally diversified stress. Simultaneous analysis of the degree of the accumulation of different physiological elements in the semen of men from polluted areas, as well as lipoperoxidation processes and reactions from oxidative enzymatic and non-enzymatic systems, will map the causal connections with the reproductive condition of particular patients.

Insufficient knowledge about the causes of impaired reproductive potential results in an inability to implement specific treatments, which is associated with a lack of positive outcomes [35]. This review allows an understanding of the role of environmental factors in shaping the body’s defense capabilities in the area of reproductive condition. In stress conditions physiological responses of the reproductive system can be estimated based on the changes in the activity of antioxidant enzymes, biochemical and structural modifications of proteins caused by oxidative stress involving products of advanced oxidation protein, assessment of oxidative stress by changing the quantity of products of advanced oxidation protein, or changes in the lipoperoxidation and pro-antioxidant mechanisms inactivation of ROS [8,11,12,14,15]. The lack of knowledge of the causes of impaired reproductive potential results in an inability to implement specific treatment, which is associated with the lack of positive outcomes (pregnancy). This review will make relevant environmental comparisons. It will allow an understanding of the importance of environmental factors in shaping the body’s defenses and capabilities in the field of reproductive condition. The results can be used in enhancing diagnosis and deciding on appropriate infertility treatment. Physiological responses in the semen and blood of patients (specified above) are indicative of changes in the reaction to stress conditions.

A further purpose of this review is to analyze the immunological mechanisms that determine male reproductive potential and the impact of environmental stress on semen quality parameters. This is of major significance since bioaccumulation of toxic metals causes oxidative stress, which negatively impacts the condition of the semen. These events lead to alterations in the activity of caspase proteins leading to apoptosis in the germ cells [8]. Most of the negative changes mentioned above result from degradation of the natural environment with toxic metals, pesticides, or chemicals used in the industry [4,6,7]. Since oxidative stress may contribute to DNA damage, the connected causes of human infertility appear at the genetic level. Mutations responsible for pathophysiological changes in the human reproductive system occur in Down syndrome (trisomy of autosome 21), Edwards syndrome (trisomy of autosome 18), Patau syndrome (trisomy of autosome 13), Klinefelter syndrome, Turner syndrome (complete or partial absence of one of the X chromosomes in all cells of the body or a portion thereof), or cystic fibrosis (mucoviscidosis) [23,36]. These mutations may create a serious, usually irreversible threat to male fertility with diverse prevalence. Simultaneous analysis of the degree of accumulation of different physiological elements in the semen of men from polluted sites will trace the causal connections listed above in parallel with the reactions of the biochemical systems and the level of elements, lipoperoxidation, and oxidative enzymatic and non-enzymatic systems. Here it is important to take account of links between environmental elements and conventional pathologies associated with male infertility in correlation with selected biochemistry (total protein, albumin, cholesterol, glucose, fructose, bilirubin, alanino-aminotransferase ALAT, aspartat-aminotransferase ASPAT, urea, enzymes (akrosine, alkaline, and acid phosphatase), and thioneins. Complementing this evaluation is the analysis of the extracellular matrix, the components of which also mediate intercellular communication through (1) binding of cytokines or concentrate them in certain locations; (2) presentation of cells; and (3) direct binding of the individual components with specific cell receptors, which causes specific changes in the cell metabolism.

This review analyzes the immunological mechanisms that determine male reproductive potential and the impact of environmental stress on semen quality parameters. The influence of chemical elements with different physiological groups on the morphometry of semen of people living in areas with varying degrees of contamination and degradation changes (acidification, salinity, increased levels of Ca, Fe, Mg, and trace elements) is discussed. Bioaccumulation of many elements causes oxidative stress, which leads to apoptosis and determines the condition of the semen. These events lead to alterations in the activity of caspases and induction of apoptosis in the germ cells. We examine the activity of antioxidant enzymes, which may differ significantly to the control group. Chemical elements, not yet analyzed in the study of infertility (Al, Ni, Cr, Mn, As, Se, Si), play an important role in the induction of immunogenetic changes and affect the activity of antioxidant enzymes. The changes may result from degradation of the environment with heavy metals, pesticides, and chemicals used in industry. These genetic mutations are responsible for the genetic pathophysiological changes (as above). Simultaneously, one of the causes of male infertility is immunogenetical change. Therefore, we should consider the cumulative impact of xenobiotics in the semen on the occurrence of mutations responsible for these diseases and disorders of spermatogenesis, in the form of the expression and deletion of genes. Previous studies give conflicting results about the effects of chemical elements on sperm. Much of the work relates to their direct impact or has been carried out on the seed derived from persons occupationally exposed [37]. This knowledge is incomplete and needs to be reviewed, but the condition of human sperm deteriorates significantly. Further research should broaden the understanding of the impact of elements on immunogenetic disorders in male infertility, both in lipoperoxidation and antioxidant activity, as well as reactions with reductases and stress proteins. This will determine the distribution of the prevalence of these changes in regions where such research has not been conducted. This will enable the mapping of the distribution of immunogenetic changes, the dangerous mutation of DNA, semen biochemical parameters, and concentrations of chemical elements in it. The results can be used in the prevention of infertility in women living in degraded areas. They will also shed light on the causes of infertility in those men who were previously fertile. Linking biochemical analysis of semen and immunogenetic changes elucidates the mechanisms and clarifies the role of heredity factors in shaping the response to environmental stress by oxidative enzyme systems. The results can be used in the diagnosis of male infertility undergoing environmental weakening. In addition, the levels of oxidative enzyme activity circuits and an analysis of the lipoperoxidation intensity and protein levels of stress can give an index of sperm health conditions in humans.

## 2. The Current State of Knowledge

### 2.1. Molecules Affecting Male Infertility

Currently, 30% of men suffer from idiopathic infertility [38]. The standard semen analysis is still the most important clinical assessment of male reproductive potential. The results of this analysis determine ejaculate capacity, sperm count, motility, and morphology. Among the basic components of the sperm plasma ions Na, K, Mg, Ca, Fe, Cu, Zn, and Se are the most significant [39]. The potassium concentration in the sperm plasma should be 27 ± 5 µmol (1.1 mg × mL^−1^). When the ratio of Na/K exceeds 1:2.5, it affects sperm motility and an increased concentration of potassium cations increases the electrical charge of the sperm cell membrane decreasing the motility of cell [40]. Each element plays a different role in the body, thus destabilizating their level has serious consequences. Ca, Mg, and other electrolytes maintain osmotic equilibrium and are involved in the transport of nutrients. Zn and Fe are involved in redox processes. Zn and Mg are stabilizers of cellular membranes and coenzymes of SOD, which prevents the harmful effects of free radicals on sperm [13,15]. Zinc, as one of the most important factors influencing male sexuality, is involved in processes of reproduction, in both hormone metabolism and sperm formation, as well as in the regulation of sperm viability and motility [14]. Zn deficiency results in decreased levels of testosterone and decreased sperm count, potency disorders, reduced sperm viability and even infertility [41]. Zinc, as an antioxidant plays an important role in the protection of spermatozoa from the attack of free radicals. High levels of Zn in the semen decrease the activity of oxygen radicals, maintaining sperm in a relatively quiet and less motile state, resulting in a lower consumption of oxygen which allows the storage of energy needed during the passage through the genital tract. Zn also has a protective effect against too high a concentration of Pb (contributing to reduction of fertility) [15]. Even with a high Pb accumulation, elevated Zn concentration has a protective effect, reducing the harmful effects of this element [42,43]. Chia et al. (2001) [44] have demonstrated a correlation between the concentration of Zn in the blood and semen plasma, and the quality of sperm from fertile and infertile men. The results showed lower Zn levels (accompanying lower morphologic parameters) in patients with impaired fertility (183.6 mg·L^−1^). In fertile patients Zn level was much higher (274.6 mg × L^−1^). Thus, Zn has a positive impact on fertility and potency through participation in spermatogenesis [44]. An important role of Zn was also described by Giller (1994) [45], indicating that semen volume decreases by 30% at a low Zn concentration. Similarly, Mohan et al. (1997) [46] have shown that men with low daily Zn intake (only 1.4 mg) displayed a significant decline in semen capacity and concentration of testosterone in serum. A relationship was also shown between the level of Zn in serum and semen in oligozoospermic infertile men, with significantly lower levels of Zn in serum and semen of men with fertility problems [46].

The second element of fundamental importance for semen quality is selenium, which occurs in high concentrations in semen and plays an important role in maintaining reproductive condition [13,14]. Selenium is an essential microelement at low levels of intake and produces toxic symptoms when ingested at level only 3–5 times higher than those required for adequate intake. Se-counteract the toxicity of heavy metals such as Cd, inorganic mercury, methylmercury, thallium and to a limited Ag extent. Although not as effective as Se, vitamin E significantly alters methylmercury toxicity and is more effective than Se against silver toxicity. Selenium can particularly counteract Hg toxicity, and is the key to understanding Hg exposure risks. Selenium compound selenide binds mercury by forming mercury selenide, which neutralizes the harmful effect of Hg. However, once that bond is made, Se is no longer available to react with selenoproteins that depend on it. Human studies have demonstrated that selenium may reduce As accumulation in the organism and protect against As-related skin lesions. Se was found to antagonize the prooxidant and genotoxic effects of As. From epidemiological point of view Se interaction with heavy metals raises a large interest. Although antagonistic influence of Se on the bioaccumulation of Hg, Cd, and As is well known, interaction mechanism between those elements in humans remain unexplained [47]. Selenium takes part in the constitution of the mitochondrial shield in sperm cells and influences the condition and function of sperm, and is effective in the treatment of impaired fertility [47]. Simultaneously, selenium as part of selenoproteins, playing a key role in defending the body against oxidative stress [48]. Phospholipid hydroperoxide glutathione peroxidase PHGPx changes the physical properties and biological activity during the maturation of sperm. In spermatids it displays enzymatic activity and is soluble, while in mature sperm it is present as an inactive and insoluble protein. Inside the mature sperm PHGPx protein constitutes at least 50% of the material of the shield [49]. However, toxic heavy metals (Cd, Pb, Hg, Ni, Cr, B, V) impair testicular function and the mechanisms of their toxic activity in the nucleus include damage of the vascular endothelium of the Leydig’ and Sertoli’ cells but these heavy metals not only damage the vascular endothelium but as stated for example, in [50,51], Cd and Pb cause an alteration in the functionality of the Sertoli cell even at subtoxic doses. Oxidative stress occurs as a result of their accumulation due to impairment of antioxidative defensive mechanisms and intensification of the inflammatory reaction leading to changes in the morphology and function of the testes [1,2,6,7,10,52,53]. The effect of these changes can be necrosis of the seminiferous tubules, which inhibits the synthesis of testosterone and impairs spermatogenesis. Short-term exposure to these metals increases the activity of SOD, CAT, GPx, and glutathione reductase GR, which is indicative of the activation of defense mechanisms and the adaptive response of cells [9,54]. 

In order to fully analyze the problem, we should distinguish precisely the functions of individual forms of GPx and their importance for the male reproductive system. Glutathione peroxidases are composed of eight forms that are distributed in different tissues with differences among species [55]. They catalyze the reaction needed to remove hydrogen peroxide H_2_O_2_ and other hydroperoxides using reduced glutathione GSH. In order to keep removing hydroperoxides, the oxidized glutathione disulfide GSSG must be reduced back to GSH by the GR enzyme using NADPH as reducing agent. There are selenium-dependent and selenium-independent GPx forms. The first group is represented by GPx1–4 and the second group by GPx5–8. GPx forms can also reduce peroxynitrites ONOO^−^, a very reactive ROS capable of harming cells promoting tyrosine nitration in proteins involved in motility and sperm capacitation [55]. Of great importance for spermatozoa is the presence of the selenoprotein phospholipid hydroperoxide GPx4 (PHGPx), a structural protein which is essential for normal formation of the mitochondrial sheath and constitutes about 50% of the sperm midpiece protein content localized in the mitochondrial helix. The need for mitochondrial PHGPx (mGPx4) to assure normal sperm function has been demonstrated in humans since infertile men have shown low sperm motility with abnormal morphology [55]. It is important to highlight that what is relevant for fertility is the ability of mGPx4 to interact with hydroperoxides to form the mitochondrial sheath during spermiogenesis and not its antioxidant activity which is less than 3% of the total PHGPx protein content in ejaculated spermatozoa. Selenium is essential to assure normal GPx4 function during spermiogenesis as it was confirmed by the presence of abnormal spermatozoa with poor motility [55]. 

The sperm chromatin formation during spermiogenesis is accomplished in part by the nuclear isoform of GPx4 (snGPx4); this enzyme mediates the oxidation of S–H groups of protamines by hydroperoxides. It is possible then that other proteins are involved in the sperm chromatin re-modelling and potential candidates are peroxiredoxins. The contribution of GPx to the protection against ROS is limited in human spermatozoa since human spermatozoa, testes, or seminal plasma lacks GPx2, GPx3, and GPx5 and GPx4 are insoluble and enzymatically inactive in mature ejaculated spermatozoa [55]. It seems that the role of GPx1 as important antioxidant enzyme is questionable because Gpx1^−/−^ males are fertile and they are not susceptible to oxidative stress and lipid peroxidation does not increase in human spermatozoa incubated with H_2_O_2_ in the presence of carmustine (GR inhibitor) or diethyl maleate (binds to GSH making it non-accessible for GPx/GR system) that affects the GPx/GR system activity [55]. 

In turn, Gladyshev et al. (2016) [56] indicates that the human genome contains genes coding for selenocysteine-containing proteins (selenoproteins). These proteins are involved in a variety of functions, most notably redox homeostasis. Selenoprotein enzymes with known functions are designated according to these ones. Selenoproteins with no known function appear to be important but require further research.

A particularly dangerous heavy metal for semen quality is lead. It is increasingly recognized that impaired fertility in men can be associated with environmental and occupational exposure to lead [10,57]. The mechanism of action of lead on male gonads is complex and includes effects on spermatogenesis, steroidogenesis, the redox system, and damage of the vascular endothelium of the gonads by free radicals, resulting in morphological changes (weight changes of the testes and seminal vesicles, their fibrosis, a reduction in the diameter of the seminiferous tubules, and a reduction in the population of reproductive cells by apoptosis) and functional changes (decreased testosterone synthesis). Lead may affect the function of Leydig’ cells impairing steroidogenesis, decreasing the levels of testosterone and worsening the quality of sperm” but this observation is valid not only for Leydig cells but also for Sertoli cells that are the sentinel of spermatogenesis [1,7,51,54]. The phenomenon of oxidative stress in animals poisoned with lead confirms an increase in lipid peroxides and decomposition of thiobarbituric acid reactive substances TBARS [58].

### 2.2. Antioxidant Mechanisms

A significant role in the pathogenesis of infertility involves redox reactions because the germ cells are capable of producing ROS. A certain physiological amount of reactive metabolites of oxygen, rising in the respiratory chain, is necessary to maintain normal sperm functionality. However, due to overproduction of ROS or the exhaustion of the compensating possibilities of antioxidative mechanisms in sperm, oxidative stress begins to increase [7,9]. Subsequently, it leads to changes in peroxidation of lipid membranes of sperm, impairing the structure of membrane receptors, enzymes, transport proteins, and leads to an increase in the level of DNA fragmentation of sperm [59,60,61]. The balance between ROS formation and the protective actions of antioxidative system is necessary to sustain normal functions of an organism [8]. The important area of influence of essential elements are metabolic mechanisms, i.e., reactions involving compounds quenching excited molecules, non-enzymatic mechanisms (ceruloplasmin, transferrin, polyamides, transitional metals, sequestration of metals, thioneins), antioxidant enzymatic mechanisms (SOD, CAT, GPx, GR, glutathione S-transferase GST, secretory phospholipase A2 sPLA2, reactions involving heat shock protein HSP, chaperones, and proteases [59,60,61]. Due to the particular sensitivity of male reproductive cells to the oxidative action of ROS, mammalian semen is equipped with a variety of enzymatic and non-enzymatic compounds, which neutralize the excess of ROS, localized in the seminal plasma and inside sperm cells [59,60,61]. A direct relationship between the SOD activity and sperm damage and sperm motility was confirmed by numerous researchers [9]. The addition of exogenous SOD to a suspension of sperm cells protected their vitality and significantly affected motility by inhibiting the destruction of biological membranes. However, some researchers could not confirm the effect of SOD on semen quality and sperm fertilizing potential [62,63].

The most effective antioxidative enzyme in sperm apart from SOD is CAT [12,13]. It was found inside sperm cells and seminal plasma, with activity significantly reduced in infertile men [64]. Another important enzyme that protects cells from the toxic effects of H_2_O_2_ is GPx. The sperm GPx is located in the mitochondrial matrix. Its activity is largely related to the level of Se in semen [13,14,15]. The important protective role of GPx in counteracting the loss of sperm motility as a result of spontaneous lipoperoxidation has been widely confirmed. Many researchers have proved the relationship between peroxidative damage of sperm and male infertility [62], because lipoperoxidation is one of the most important processes related to the action of ROS. The accumulation of damaged lipid molecules lowers the fluidity of biological membranes and the structural damage of membranes has a direct impact on their receptor and transport functions [9].

### 2.3. Genetic Effects

The accumulation of heavy metals in an organism and the impact of free radicals can cause immunogenetic disorders, chromosomal aberrations and consequently lead to serious genetic defects, causing infertility include numerical and structural aberrations that may affect autosomes or sex chromosomes [65,66,67,68]. Chromosomal aberrations appear in 7% of infertile men, that is 30 times more frequently than in the general population [69,70]. The most common chromosomal cause of male infertility is Klinefelter syndrome (>4%) [71]. In this disease, similarly to Turner syndrome, partial fertility is maintained only in mosaicism [66,72]. In Klinefelter syndrome changes in nuclear structure leading to infertility may be a result of the presence of two alleles of many genes associated with the X chromosome, which typically operate on the principle of disomy and do not undergo inactivation during lyonization of extra chromosome. In 15% of males with azoospermia and 5% with oligozoospermia display an abnormal karyotype [71,73]. Another cause of male infertility is microdeletions of the Y chromosome or aberrations and mutations of genes responsible for male sexual development, e.g., located in the short arms of the Y chromosome in the region Yp11.2 (the Yp11.2 region containing the amelogenin gene on the Y chromosome *AMELY* locus). The amelogenin gene on the Y chromosome, *AMELY*, is a homolog of the X chromosome amelogenin gene *AMELX*, and the marker is employed for sexing in forensic casework, *SRY* gene (a sex-determining gene on the Y chromosome). *SRY* gene, as a sex-determining gene on the Y chromosome in mammals that determines maleness and is essential for development of the testes; testis-determining factor TDF, known as sex-determining region Y SRY protein, is a DNA-binding protein (known as gene-regulatory protein/transcription factor) encoded by the *SRY* gene that is responsible for the initiation of male sex determination in humans). Another reason for male infertility is the partially symptomatical form of cystic fibrosis, responsible for 60% of the so-called obstructive azoospermy [23,36]. The true symptomatic form of cystic fibrosis is the result of mutations in the *CFTR* gene and in 95% cases of men leads to infertility [74,75].

The current state of knowledge about male fertility conditions does not give clear and unambiguous answers to the cause of the growing problem of infertility. We cannot determine unambiguously which environmental factors have the greatest impact on human fertility. It is, therefore, necessary to continue research in the field of concentration of elements, oxidative enzyme activity, and the incidence of immunogenetic disorders in the seed. These analyses are a benchmark in project design, making it possible to verify the views on the impact of environmental stressors on male fertility. The results of these studies can be applied in the prevention of infertility and contribute to the development of new diagnostics.

## 3. Potentially Harmful Factors in the Natural Environment: From Heavy Metals to Domestic Dust

Toxic heavy metals are one of the main sources of causative male infertility. From the beginning of their activities at the cellular level, they generate a series of reactions that destabilize normal processes within the cell organelles. Such a permanent and deepening interaction causes a gradual shift of the metabolic pathways and biochemical processes of the cell, including a change in normal transcription and translation in the nucleus. This ultimately generates genetic polymorphisms, responsible for the formation of changes in the male reproductive condition [1,2,52]. Among other destructive factors generally present in the environment we can enumerate combustion products, traffic fumes, dioxins, polychlorinated biphenyls, pesticides, food additives, and persistent pollutants, such as DDT [4,5,6,53]. A separate group includes potentially harmful factors that remain under human control, such as smoking, obesity, and a sedentary lifestyle. All of these can play the role in lowering reproductive condition resulting in decreased sperm counts, even among very young men [6]. Certain metals that we are exposed to almost every day, e.g., Cu, Pb, Cd, or Mo influence reproductive hormone levels (such as testosterone). Simultaneously, Meeker et al. (2010) [2] proved that certain interactions between metals in humans can modify serum testosterone level. Based on analysis of 219 relatively young men, researchers observed a 37% reduction in testosterone levels in the case of men with high Mo and low Zn concentrations in blood. Additionally, they observed higher Cu and Cd levels accompanying low Zn concentration among smokers. However, Buck et al. (2012) [53] broadened their investigation to both men and women reproductive conditions with environmental Cd and Pb exposure. This study sampled over 500 couples willing to have a child. The researchers measured the time to pregnancy in each case, and included daily questionnaires, filled by couples, about their lifestyles. The investigation encompassed two regions, selected to ensure a range of environmental exposures to heavy metals. Their results confirmed that environmentally relevant concentrations of blood Pb and Cd make time to pregnancy longer. Thus, couple fecundity decreased with more frequent exposures to toxic metals.

Generally, toxic metals are considered as strong oxidative stress inducers and endocrine disruptors in humans, and are particularly harmful to the testis. Similarly to Pb, Hg, and estrogenic compounds, Cd can seriously disrupt the functionality of the testis and, as a consequence, reduce sperm count and quality. Siu et al. (2009) [52] enquired how exactly Cd damaged the testicles and stated that the disruption of the blood-testis barrier applied to complex pathways of signal transduction and signaling molecules like kinase p38 (human mitogen-activated protein kinase 14/p38 alpha (active enzyme recombinant, human protein kinase p38; stress-activated protein kinase). Cadmium exposure appears to be a potential risk factor for testis injury via oxidative stress stimulation, endocrine destabilization, and certain interactions with protective elements, such as Zn [52]. Moreover, in the study conducted by [1], researchers expanded the pool of analyzed metals and testified to the environmental toxicity of Cd, Cr, Pb, Hg, As, and especially Mo. The authors linked semen quality with estimated blood concentrations of the enumerated elements. That investigative group involved over 200 men (patients from infertility clinics). The most surprising finding concerned molybdenum. Researchers observed a dose-dependent relationship between Mo and a decrease in sperm concentration and motility. Based on this result we could add molybdenum to the list of potential threats to male fertility. However, the toxicity of Cd, As, Pb, and Hg and their influence on a decline in semen quality was more obvious [1]. Simultaneously, Vaiserman (2014) [4] mentions that endocrine-disrupting chemicals are invariably present in the environment of industrialized societies. The list includes dioxin, dioxin-like compounds, phthalates, polychlorinated biphenyls, pharmaceuticals, agricultural pesticides, and industrial solvents. Their destructive role in chronic endocrine pathologies is doubtless and leads to negative estrogenic and anti-estrogenic activity. However, the damage is particularly detrimental at a genetic level, causing a threat to the normal development of the organism, which has been widely analyzed in animal models, e.g., exposure to dioxins disrupts the expression of genes involved in extra-cellular matrix remodeling in the cells of the cardiac muscle. Methoxychlor alters the methylation pattern of paternally and maternally imprinted genes in the sperm of mice offspring. Bisphenol A causes hypermethylation of the estrogen receptor promoter region in the adult testis of rats in addition to modifying hepatic DNA methylation [4]. Despite the fact that in Vaiserman’s [4] study the negative effects mentioned were verified mostly on rats and mice, the author suggested that a similar impact on people was of high probability. He highlighted that in the last number of decades the endocrine condition of humans has decrease seriously, subsequently worsening reproductive condition. In both problems the most serious changes occur due to toxic exposure in the prenatal period or early childhood, resulting in defective development of the organism in later years. These statements agree with [5], who also considered long term exposure to herbicides, formamide, antimetabolites, fungicidal preparations, dyes, and obviously toxic metals (Cd, Pb, Cr, Ni) as harmful factors that considerably worsen the quality of sperm.

If the realization that heavy metals and certain chemicals decrease human reproductive condition still does not bother us, then there is an example of a further disruptor from our close surroundings. Meeker and Stapleton (2010) [3] proved that even house dust can modify levels of reproductive hormones and diminish sperm quality. Researchers analyzed organophosphate compounds, commonly used as additive flame retardants and plasticizers in popular domestic materials. Semen parameters and reproductive hormone levels were measured in 50 men from infertility clinic who had frequent contact with these materials. They concluded that organophosphate compounds from typical domestic equipment (contained in house dust) may not only alter certain hormone levels (such as prolactine or thyroxine), but also decrease sperm concentration by as much as 19% [3].

### 3.1. Environmental Pollutants and Oxidative Stress

Oxidative stress is a damaging process that happen when there is an excess of free radicals in the body cells. The body produces free radicals during normal metabolic processes. Intense oxidation can damage cells, proteins, and DNA, which can contribute to aging. Disturbances in the normal redox state of cells can cause toxic effects through the production of peroxides and free radicals that damage all components, including proteins, lipids, and DNA. Oxidative stress from oxidative metabolism causes base damage, as well as strand breaks in DNA. ROS and free radicals are generally known to be detrimental to human health. A large number of studies demonstrate that, in fact, free radicals contribute to initiation and progression of the changes in genetic material, i.e., genetic polymorphisms [8]. Oxidative stress happens when the balance between peroxidation and anti-oxidation is disturbed, i.e., when the production of ROS exceed cellular concentrations of small molecular antioxidants or activity of antioxidative enzymes [8]. Researchers widely consider ROS as a source of dangerous reactions, uncontrolled and harmful to structures at a molecular level [11,12,13]. As a proof Bartosz (2009) [8] enumerates several negative effects of ROS activity (degradation of collagen, depolymerization of hyaluronic acid, oxygenation of hemoglobin, inactivation of enzymes and transport proteins, lipid peroxidation in cellular membranes, damage to chromosomes, and breakages in DNA). In the face of so many threats, it is valuable to know precisely how ROS comes about. Bartosz (2009) [8] identified several factors that stimulate the formation of ROS (ionic radiation, sonication, UV radiation, oxygenation of reduced forms of molecular components of cells, oxygenation of xenobiotics, photoreduction, and oxygenation of respiratory proteins).

### 3.2. Intensification of Oxidative Stress due to Pollution—Influence on Human Fertility

The close relationship between environmental pollution and oxidative stress is central to understand why human fertility has decreased in past decades, because the most environmental toxicants induce ROS, causing oxidative stress [7]. In the human reproductive system, the testes are especially susceptible to destructive changes due to this phenomenon. The after-effects are often irreversible and include a decline in testosterone levels, disorders in spermatogenesis, and eventually infertility. Certain physiological levels of ROS are even necessary for the proper course of spermatogenesis. However, an excess of reactive oxygen radicals, formed due to environmental pollutants, destroy testicular functionality and manifest as a diminished sperm count and quality. Among toxicants inducing apoptosis in germ cells, Mathur and D’Cruz (2011) [7] have singled out methoxychlor which decreases the levels of anti-oxidative enzymes in testicles, especially in the mitochondrial and the microsomal fractions of testis. Dichloro-diphenylo-trichloro-ethane DDT metabolites, on longer exposure, cause incremental changes in lipoperoxidation and a decrease in enzymatic antioxidants such as SOD or GPx in the testis. Exposure to certain fungicides have been found to contribute to reduced prostate mass and decreased sperm count, as well as induced impairments in expression of apoptosis-related proteins such as p51. Other enumerated chemicals such as pesticides, bisphenol A and certain herbicides also damage testicles and interrupt spermatogenesis through oxidative stress stimulation [7]. Therefore, many substances that humans associate with in everyday life are, in truth, very dangerous pro-oxidants and stimulants of uncontrolled ROS formation in several body systems. Data by Agarwal et al. (2014) [9] found similar conclusions; they assert that about 15% of couples trying to conceive are struggling with infertility. Male factors can be the reason for nearly half of such cases. Oxidative stress and overproduction of ROS damage DNA, proteins, and lipids, change the functionality of enzymes and, finally, cause cell death. Like Mathur and D’Cruz (2011) [7], Agarwal et al. (2014) [9] also affirm that certain levels of ROS are necessary for correct fertilization. In normal conditions and controlled concentrations, ROS regulate sperm maturation, stimulate signaling processes and more. However, in uncontrolled ROS overloading, there is a risk of infertility. They suggest that impairments in sperm cells arise via induction of per-oxidative damages of sperm plasma membranes (per-oxidation of lipids), as well as DNA breakages. The best way to minimize the negative effects of ROS excess is to eliminate as many factors as possible. Cessation of smoking, discontinuation of alcohol abuse, a reduced-fat diet, physical activity, and antioxidant intake (supplementation of diet with carotenoids or vitamins C, E) constitute simple tactics against oxidative stress, which patients can initiate even on their own. Thus the problems of oxidative stress and ROS overproduction may be significantly reduced by reasonable changes in lifestyle. On the other hand, routine estimations of semen ROS levels should become a standard procedure in the diagnosis of male fertility [9].

Elucidation of the destructive impact of oxidative stress and factors that stimulate the phenomenon are well presented in the studies conducted by Al-Attar (2011) [10]. He provided mice drinking water with a mixture of Pb, Hg, Cd, and Cu. After seven weeks, he assessed renal function by measuring the concentrations of creatinine, urea, and uric acid. Furthermore, he measured levels of antioxidants, including glutathione GSH and SOD in kidney and testicles. Compared to the control group (mice drinking water without heavy metals) the experimental group had considerably increased creatinine (by 152%), urea (by 83%), and uric acid (by 65%). Decreases of anti-oxidative enzymes, both in kidney and testis were significant (glutathione: 28% in kidney, 24% in testicles; SOD: 40% in kidneys, 27% in testis). Moreover, in histological examination of the testis of mice exposed to heavy metals, Al-Attar (2011) [10] noted degenerative changes in the seminiferous tubules leading to disruption of spermatogenesis. In a separate experimental group the diet was supplemented with vitamin E [10], noting insignificant changes in renal parameters and a considerably smaller downgrade in testicular anti-oxidative enzymes due to the heavy metals. Thus, research demonstrated not only a negative effect of oxidative stress, but also the positive anti-oxidative potential of vitamin E in a daily diet.

### 3.3. Tactics against Oxidative Stress—Antioxidative Diet

The reduction in oxidative stress markers found by [10] explored only one of several tactics which can be deployed in the fight against uncontrolled ROS. Ruder et al. (2008) [11] explored the after-effects of oxidative stress in female infertility. Researchers suggest that lifestyle and diet, rich in antioxidants, during pregnancy also play a critical role in reproductive success. They found that high oxidation levels increase the risk of disorders during successive stages in pregnancy. On the contrary, antioxidants intake, even in the simplest form, by eating fruits or vitamin supplementations, minimizes the threat of pregnancy loss. In the case of male fertility, it is valuable to know which metals bring positive effects to the reproductive condition. One of the most important chemical elements with anti-oxidative properties is zinc. It protects sperm cells against ROS, contributes to the formation of semen and stabilizes the levels of reproductive hormones (such as testosterone) and, in general, lengthens the vitality of sperm cells [14]. Therefore, zinc is widely considered as an effective antioxidant. Oteiza (2012) [76] highlighted the beneficial Zn properties of in reducing oxidative stress. It maintains the cell redox balance, regulates oxidants production, contributes to the repair of cell damage, and regulates the metabolism of glutathione and conditions of redox signaling. Furthermore, Zn mediates in the induction of Zn-binding protein metallothionein, preventing overproduction of ROS [76]. An important beneficial element is selenium, which favors the functional efficiency of sperm cells and, as a consequence, increases semen quality [14,77]. Indeed, both elements (Zn, Se) are the molecular components of important anti-oxidative enzymes. Zn is present in SOD type 1 and 3 (as well as Cu) and Se is a component of GPx. These facts clearly demonstrate their antioxidative significance [8]. Additionally, Atig et al. (2012) [14] compared Zn and Se levels in semen samples from fertile and infertile patients. Compatible with expectations, fertile men’s sperm showed higher levels of these elements compared to infertile patients. Zinc exhibits positive and significant correlations with sperm motility and sperm count. Selenium is also significantly correlated with semen motility. Selected parameters of anti-oxidative response, such as the concentration of glutathione enzymes and the quantity of malondialdehyde MDA, a lipoperoxidation end product, were also analyzed. Glutathione enzymes were considerably decreased in infertile semen and there was a greater amount of MDA in sperm from infertile patients. On the contrary, fertile semen show high levels of glutathione enzymes and only small amounts of lipoperoxidation products. Even more, researchers confirmed a positive correlation between glutathione enzymes and sperm motility. On the contrary, MDA was negatively associated with sperm motility and concentration, as well as positively correlated with the percentage of abnormal sperm. On this basis, the authors concluded that a serious decrease in seminal antioxidants (such as Zn, Se, as well as glutathione enzymes) favors the risk of impairments in sperm quality. Additionally, increased MDA reflects a diminished sperm quality and reproductive condition [14].

Zini et al. (2009) [12] stated that the sperm of infertile men contains considerably more DNA damage than in the case of fertile patients. Therefore, the authors analyzed the potential of antioxidant therapy. They found that dietary antioxidants can efficiently reduce sperm DNA damage, especially in high levels of DNA fragmentation. In their opinion, the risk of ROS overproduction is connected with unsaturated fatty acids in sperm plasma membranes. These acids are necessary for membrane fluidity, but also predispose it to free radical attacks. On the other hand, semen contains certain levels of anti-oxidative enzymes (SOD, CAT, GPx), as well as non-enzymic antioxidants (vitamin C, E, lycopene, or l-carnitine). Accordingly, researchers proved that dietary supplementation of antioxidants (e.g., vitamin C oral intake) may cause positive effects in the improvement of sperm integrity and lowering oxidation levels. However, Walczak-Jędrzejowska et al. (2013) [13] described the destructive effects of oxidative stress on sperm cells including a decrease in activity of anti-oxidative mechanisms, damage to DNA and accelerated apoptosis. As a consequence they found a diminished number of sperm cells and their reduced motility. They highlighted that the large endogenous sources of reactive forms of oxygen in semen are white blood cells and immature sperm cells. This study emphasizes the physiological role of ROS in sperm maturation, but for the same reason any infection or inflammation process in the body could be considered as a moderator of oxidative radicals. However, unfavorable environmental factors may also initiate the analogous problem. Walczak-Jędrzejowska et al. (2013) [13] further widened the list of potentially beneficial antioxidants, adding vitamins A and B, coenzyme Q10, carotenoids, and carnitine to the known list including glutathione, Zn, Cu, Se and SOD, CAT, and GPx. Explaining the role of vitamins E and C in the defense against oxidative stress, it can be concluded that vitamin E reduces lipoperoxidation and mainly protects sperm cell membranes, while vitamin C, preventing sperm DNA damage, is a very abundant seminal antioxidant, since it is present in concentrations about 10 times higher in seminal plasma than in blood serum. They strongly recommend the initiation of antioxidant therapy in cases of men with fertility problems. Additionally, Mier-Cabrera et al. (2009) [78] compared the levels of oxidative stress markers and concentrations of anti-oxidative enzymes among women with a high antioxidant diet and a normal diet. After four months of observation, in the group on the anti-oxidative diet, the researchers noted an increase of vitamin levels (A, C, E), as well as considerable growth in activity of SOD and GPx. Furthermore, the levels of MDA and lipid hydro-peroxides (oxidative stress markers) were relatively low in this group. Conversely, in the case of women on a normal diet there was no improvement in anti-oxidative parameters or decrease in oxidative stress markers. Thus, supplementation of the daily diet with certain antioxidants (vitamins A, C, E, or Zn) may be a simple way to overcome oxidative stress on our own. Rink et al. (2013) [79] decided to check in practice how the recommended intake of fruits and vegetables (five times a day) influenced oxidative and anti-oxidative parameters. They selected 258 pre-menopausal women, observed their diet and measured pro- and anti-oxidative parameters over a period of about two menstrual cycles. Particularly important parameters were the erythrocyte activity of SOD and GPx. They noted that eating fruits and vegetables five times a day, over a longer period, considerably diminished oxidative stress (levels of lipoperoxidation markers) and improved antioxidant status (high levels of antioxidative enzymes, as well as non-enzymatic antioxidants).

Summarizing, Aitken and Roman (2008) [15] considered oxidative stress as a major factor in the etiology of male infertility. Similarly to the previously quoted research, lipoperoxidation and DNA fragmentation were considered as the most serious damage, caused by ROS in sperm cells. Furthermore, in the testicles, oxidative stress may destabilize the process of differentiation of spermatozoa. They identified and characterized the basic anti-oxidative defense line, e.g., they noted that all three types of SOD are found in the testicles. Type I (cytoplasmic) containing Zn and Cu ions, type II (mitochondrial) with Mn and, finally, type III (extra-cellular) containing Cu and Zn. There are also various isoforms of GPx located in mitochondria and the nucleus, particularly in differentiating semen. Researchers emphasize the relationship between the activity of glutathione enzymes and the presence of selenium (lower concentration of Se is connected with a decrease in activity of GPx). Among non-enzymatic antioxidants researchers listed the essentials Zn (interrupting lipid peroxidation by displacing from catalytic sites such metals as Fe and Cu and attenuating damage in sperm DNA caused by Pb or Cd), vitamin C or E (supporting the maintenance of spermatogenesis and testosterone production), as well as melatonin and cytochrome C. Melatonin is an especially valuable protector from oxidative stress due to readily crossing the blood-testis barrier, while cytochrome C assists in the elimination of damaged germ cells [15]. On the other hand, Zareba et al. (2013) [16] analyzed the influence of regular carotenoid intake in the improvement of sperm quality in 189 young, healthy men. Researchers measured such parameters as semen volume, total sperm count, motility, and morphology. After a period on a high-antioxidant diet, they found that beta-carotene and lutein intake increased sperm motility. Lycopene improved semen morphology and a longer application caused a greater amount of morphologically normal sperm. Additionally, a healthy lifestyle (regular physical activity, non-smoking) favors assimilation of antioxidants (such as vitamins C, E, A, and carotenoids). On the contrary, the intake of alcohol or caffeine was negatively associated with antioxidants assimilation, e.g., caffeine decreased the assimilation of vitamin C [16].

## 4. Genetic Reasons for Spermatogenesis Disturbances: Impairments on Chromosomes Y and 7

We are currently conducting experimental studies of male infertility determinants and we found (demonstrated) that external environmental factors and so-called internal (according to World Health Organization WHO criteria) are closely related to each other. At the same time, these detailed factors generate specific changes in genetic material (i.e., genetic polymorphisms), which are just the direct cause of male infertility. Simultaneously, the review presented above clearly explained that certain factors (environmental, artificial, or just connected with individual lifestyle) may considerably depress the human reproductive condition. Most of these factors, especially heavy metal ions, chemical compounds, and active organic residues, act by stimulating overproduction of ROS. Additionally, oxidative stress is the main reason for spermatogenesis disturbances. Many authors assert that long-lasting oxidative stress seriously damages human DNA [12,13,15]. Furthermore, genetic factors are considered responsible in at least 10–15% of cases of male infertility [80]. Therefore, it is necessary to analyze external and internal environmental genetic reasons for male infertility, as aside from the most common phenotypes.

Azoospermia is defined as a condition where a man has no measurable level of sperm cells in the semen [81]. There are various reasons for this condition, including underdevelopment of the testicles, obstruction of the spermatic ducts or, a typical genetic cause, deletions in the AZF region of chromosome Y [36]. Additionally, cystic fibrosis is an autosomal recessive disease, common in Caucasian races (with frequency of occurrence of 1/2500 live births). The genetic reasons for cystic fibrosis are mutations in the *CFTR* gene on chromosome 7. The most common mutation is the deletion of three nucleotides resulting in the loss of phenylalanine in position 508 of the protein (F508del). Approximately 70% of cases are determined by this mutation [21,22]. The manifestation of cystic fibrosis results in the production of a thick, sticky mucus in all organs containing mucous glands, coupled with pathological changes in the respiratory system (recurring pneumonia, bacterial infections) and the alimentary system (cholelithiasis, clogging of salivary glands). In the reproductive system cystic fibrosis causes an accumulation of mucus in the spermatic ducts and, as a consequence, their total obstruction [23].

### 4.1. Microdeletions in the Azoospermic Factor AZF Region

The first reported association between Y chromosome deletions and abnormal spermatogenesis was reported in 1976 by Tiepolo and Zufardi [82]. The AZF region (called azoospermia factor) was described as located in the long arm of the human Y chromosome (Yq11) and consists of the three genetic domains azoospermic factor of region “a” AZFa (proximal), azoospermic factor of region “b” AZFb (intermediate), and azoospermic factor of region “c” AZFc (distal). AZFc is one of the most genetically dynamic regions (c) in the human genome, possibly serving as counter against the genetic degeneracy associated with the lack of a partner chromosome during meiosis. Since the AZF region contains genes essential for proper spermatogenesis, microdeletions in the range of particular domains were implicated in spermatogenic impairments [17,18,83,84]. Many authors consider not three but four AZF domains as associated with spermatogenesis disturbances. This classification is based on structural observation which found that AZFb and c partially overlapped. This region of overlap is now called azoospermic factor of region “d” AZFd and is located between AZFb and AZFc [84,85]. Depending on the location of the AZF microdeletion, the phenotypes vary from mild (<15 × 10^6^ spermatozoa × mL^−1^) or severe (<5 × 10^6^ spermatozoa × L^−1^) oligozoospermia to azoospermia (complete lack of sperm cells in ejaculation) [19,81]. The complete deletion of AZFa leads to azoospermia and Sertoli Cell Only Syndrome SCOS while microdeletions in AZFb are connected with azoospermia due to the failure of sperm maturation usually at the spermatocyte/spermatid stage (subsequently there is practically no sperm in the testis of such patients). The AZFc deletion is connected with various possible seminal damages, but usually in patients a small amount of semen is present in the ejaculate (up to 60% of cases). Such patients are classified as azoospermic or oligozoospermic [18,83]. Microdeletions in AZFd lead to a mild form of oligozoospermia and abnormal sperm morphology [35,84]. Among infertile men the prevalence of AZF microdeletions is estimated at 7–8%, with a wide variation across populations [81,86]. Massart et al. (2012) [86] estimated the world frequency of Yq microdeletions among infertile men at 7.4%, based on over 90 articles, including over 13,000 patients suffering from infertility in different populations. Some researchers stated that the prevalence of Yq microdeletions is higher in azoospermic men (9.7%) than in oligozoospermic (6.0%). Moreover, they estimated the average frequency of microdeletions in particular domains. Complete deletion of AZFa is rare, responsible for a maximum 7% of all AZF incidents, while microdeletions in AZFb are twice as frequent, i.e., accounting for 14% of cases. AZFc impairments are considered the most common accounting for 69% of all AZF microdeletions. The rest of the pool (10% of AZF cases) is made up of a mixture of microdeletions in several domains, such as AZFa+b, AZFb+c, or AZFa+b+c [86]. Amongst the various AZF genes, the DAZ gene family (essential for regulation of spermatogenesis) is reported as the most frequently deleted AZF candidate [35]. DAZ genes are located within the AZFc domain, which undergoes deletion most commonly [36]. However, the exact frequency of AZF microdeletions among infertile men is difficult to determine. The differentiation in prevalence among patients from various populations ranges from 1% to as much as 35%. It has been estimated as 15% in Spain and Italy, 1–4% in Germany and France, 10% in China and the USA, 8% in India and Netherlands, and 12% in Tunisia and Mexico [20,80,83]. Furthermore, ethnic mutability in modern populations tends to increase the incidence making the matter more complex [81,86]. As a result, research teams usually concentrate on respective regions of the world and individual populations.

Wang et al. (2010) [19] generally regarded chromosome Y as structurally variable and susceptible to duplications, inversions and deletions. As it was mentioned, microdeletions in the AZF region are quite frequent among infertile male patients leading to spermatogenesis disruption (for instance as a consequence of sperm arrest). Therefore, Wang et al. (2010) [19] investigated the frequency of AZF microdeletions in infertile men from Northeastern China. In the experimental group, which consisted of 305 patients, researchers diagnosed 28 cases of AZF microdeletions. Their frequency was in following order; AZFc+d, AZFc, AZFb+c+d, with AZFa being least common. These authors also stated that the observed frequency of AZF microdeletions in the region they investigated, paralleled the levels in neighboring regions of the world. Additionally, Balkan et al. (2008) [35] conducted a similar analysis with 80 infertile men from Southeast Turkey. Most of them were azoospermic (54) and oligozoospermic (25). The researchers found chromosomal abnormalities in nine cases. Among them, Klinefelter syndrome was diagnosed in seven patients. Two patients had balanced autosomal rearrangements. In addition, AZF microdeletions were localized in one patient (with apparently normal karyotype and azoospermia) both in the AZFc and the AZFd regions [35]. These authors did not observe any cases of impairments in the AZFa or AZFb domains. Simultaneously, [80] examined the frequency of AZF microdeletions in a central Indian population: 156 patients (95 with oligozoospermia and 61 with azoospermia). Thirteen showed deletions in the AZF region (eight from the azoospermic subgroup and five from the oligozoospermic subgroup). They reported the most frequent deletions in the AZFc, followed by the AZFb and AZFa regions. Küçükaslan et al. (2013) [84] focused their study on a similar population which included 3650 infertile Indian men (combining patients from their own experimental group with other described cases of Yq deletions in India). They reported 215 cases with Yq microdeletions. Impairments in the AZFc domain predominated both in oligozoospermic and azoospermic patients. However, the frequency of AZF microdeletions differed significantly between regions in India.

Hellani et al. (2006) [87] claimed that among the genetic reasons for spermatogenesis disruption microdeletions in chromosome Y represent one of the most common causes. They conducted an analysis of the frequency of AZF microdeletions in the Kingdom of Saudi Arabia. Among 257 male patients with various forms of spermatogenesis disturbances (from oligozoospermia to azoospermia), 10 had chromosomal rearrangements, while in the remaining 247, eight men had microdeletions in AZF. Six of them in AZFc, one in AZFb, and one in AZFa+c. Moreover, Khabour et al. (2014) [20] identified several reasons for male infertility, such as hormonal abnormalities, the presence of antispermic antibodies, erectile disfunction, testicular cancer, and exposure to radiation and chemical agents. Thus, infertility is usually connected with complex etiology. They mentioned that nearly 40% of cases of male infertility are idiopathic. Amongst genetic causes, they still place chromosomal abnormalities as the number one reason for infertility (e.g., aneuploidy in sex chromosomes), however, AZF microdeletions are, in their opinion, the second most common reason. Therefore, similar to previously quoted studies, Khabour et al. (2014) [20] analyzed the frequency of AZF microdeletions, this time in the Jordanian population. His analysis included infertile men with azoospermia and oligozoospermia. They found partial AZF deletions in three patients from the azoospermic subgroup, two with microdeletions in the AZFc domain and one in AZFb+a+c domains.

The majority of authors agree that deletions in chromosome Y, particularly in the AZF region are one of the most important factors causing spermatogenesis disturbances and male infertility. The majority of analyses confirmed that microdeletions in AZFc are the most frequent and mostly connected with spermatogenic failure. Alongside karyotype abnormalities (affecting about 15% of azoospermic and 6% of oligozoospermic patients), AZF microdeletions are widely considered as the second most common genetic reason for male infertility [17,18,20]. It is more and more accepted to use AZF microdeletions as a specific marker of male infertility. Immense advantage results from the fact that small Yq deletions cannot be visualized in standard karyotype analysis. Therefore, their detection may explain the reason of infertility among men with apparently normal karyotypes [17,18,87]. The detection of AZF microdeletions is also recommended prior to assisted reproduction procedures such as intra-cytoplasmic sperm injection ICSI or testicular sperm extraction TESE. It is critically important in the case of patients with AZFc microdeletions, which are able to produce a certain amount of normal sperm during ejaculation and may achieve reproductive success using these techniques. Since AZF microdeletions transmit to male offspring, such patients should be advised of the possible consequences of assisted reproduction [35,83,84]. Therefore, screening for AZF microdeletions is becoming one of the first steps in diagnostics of potential causes of male reproductive problems. Typical AZF analysis includes DNA extraction (usually from peripheral blood) analyzed by polymerase chain reaction PCR-multiplex procedure with special markers for AZF microdeletions, i.e., sequence-tagged sites STS [80,85]. Ultimately, the detection of AZF microdeletions can be useful both in explaining idiopathic cases of male infertility as well as in genetic consulting prior to assisted reproduction [87]. In the case of idiopathic infertility (30–40% cases of male infertility) a genetic cause is a usually suspected [35]. Therefore, the analysis of the AZF region of the Y chromosome is necessary for accurate diagnosis.

### 4.2. Cystic Fibrosis and Congenital Bilateral Absence of the Vas Deferens

As mentioned previously, cystic fibrosis may also play a critical role in infertility (due to complete obstruction of spermatic ducts). As well as the congenital bilateral absence of the vas deferens CBAVD, Klinefelter and Kallmann syndromes are all connected with spermatogenesis disruptions [36]. CBAVD is manifested as aplasia of the spermatic ducts. Similarly to cystic fibrosis, CBAVD is caused by mutations in the *CFTR* gene. As a consequence it has been considered as an expression of cystic fibrosis or as a separate disease [21,23,24], estimated that CBAVD appeared in 99% of adult men with cystic fibrosis. However, in their analysis they concentrated on congenital bilateral absence of the vas deferens among young boys with cystic fibrosis aged 2–12. In the examined group which consisted of boys there were two subgroups identified. The first one contained children with pancreatic insufficiency and the second contained pancreatic sufficient boys. In five boys with congenital bilateral absence of vas deferens CBAVD seminal vesicles were observed. Furthermore, testicular micro-lithiasis was diagnosed in the subgroup with pancreatic insufficiency. They concluded that genital impairments in cystic fibrosis may appear at a very early age. Such manifestations were less common in young patients than in adults and appeared more frequently among youngsters with pancreatic insufficiency [24]. Moreover, Xu et al. (2014) [25] consider CBAVD as an abnormality in the male reproductive system, directly connected with the obstruction of sperm outflow into the urethra. On the basis of data review, the authors concluded that this impairment is responsible for 2% of cases of male infertility. They assert that in about 97% of male patients with cystic fibrosis, CBAVD is also diagnosed (comparable to that estimated by [24]). This fact is explained by the common genetic background, both for cystic fibrosis and CBAVD, namely mutation in the *CFTR* gene on chromosome 7. Abnormalities in the expression of *CFTR* also contribute to reduced functionality of the respiratory system, sweat glands, and reproductive system (a classical set of anomalies in cystic fibrosis patients). Thus, Xu et al. (2014) [25] confirmed the relationship between the most common variations of *CFTR* and CBAVD. Their results also suggest that certain *CFTR* variations are responsible for the more frequent occurrence of CBAVD in some populations, e.g., variation 5T creates a threat of CBAVD among French, Spanish, Japanese, Chinese, Iranian, Indian, Mexican and Egyptian populations, whilst variation of deltaF508 creates a risk for Slovenians, Canadians, Iranians, and Egyptians.

Simultaneously, Du et al. (2014) [88] considered CBAVD as a reason of nearly 6% of cases of obstructive azoospermia. Furthermore about 75% of CBAVD cases were direct manifestations of *CFTR* mutations F508del, 5T, and R117H (types of mutations in CBAVD). Accordingly, the observation that mutations of the *CFTR* gene (F508del, as well as 5T allele of the intron 8 of *CFTR*) are connected with CBAVD parallels with the results of [25]. Additionally, variations of the TG-repeats (TG13T5 or TG12T5; type of mutations in CBAVD), in their opinion, also play a part in the manifestation of CBAVD [88]. However, Massart et al. (2012) [86] noticed that about 88% of patients with two *CFTR* mutations carry severe mutation transformed to a mild mutation (respectively no *CFTR* function or residual *CFTR* function), whilst only 12% carry two mild mutations. Bareil et al. (2007) [89] investigated the connections between CBAVD and cystic fibrosis, while checking the participation of polymorphisms of transforming growth factor TGFB1 and endothelin receptor type A EDNRA in CBAVD manifestation. They suggest that both factors contribute to the lung manifestation of cystic fibrosis. This confirmation of the contribution of TGFB1 or EDNRA to CBAVD could point to another common link between cystic fibrosis and CBAVD. Du et al. (2014) [88] analyzed DNA samples from 80 patients with CBAVD (experimental group) and 51 healthy men as a control group. They indicated that polymorphism of the EDNRA may be connected with the manifestation CBAVD. Additionally, Havasi et al. (2010) [90] stated that nearly 98% of men with cystic fibrosis also suffered from CBAVD and infertility, while in 80–97% of CBAVD cases the disease were caused by at least one defective *CFTR* allele and in 50–93% of cases they detected two abnormal *CFTR* variants. These data support the statements of Bareil et al. (2007) [89]. 

Moreover, Noone and Knowles (2001) [22] characterized cystic fibrosis as a recessive genetic disease caused by mutations on both *CFTR* alleles. They described a standard set of symptoms including sino-pulmonary disease, male infertility, pancreatic exocrine insufficiency, and abnormal sweat electrolytes adding that the classic form of cystic fibrosis can be easily diagnosed in early life by conducting a sweat test (detection of abnormal chlorine and sodium levels) or by *CFTR* mutation analysis. They found that two-thirds of patients in the USA carry at least one copy of the deltaF508 mutation (one of the most common mutations in cystic fibrosis). However, they explain that the spectrum of possible impairments in the *CFTR* is extremely variable and, therefore, many phenotypes are described depending on the severity of the mutations involved (severe, mild, or atypical sets of symptoms). Therefore, about 7% of cystic fibrosis patients are still not diagnosed by the age of 10 or 15 years [22]. These researchers more recently ascribed the *CFTR* gene to the production of a trans-membrane protein securing epithelial cell functionality, especially in ion and water transport. Thus, the formation of thick, sticky mucus in the respiratory, alimentary, and reproductive systems is directly connected with inappropriate water distribution and chloride deficiency (major contributors to mucus consistency). In normal conditions the excess mucus is easily eliminated, while in cystic fibrosis the sticky mucus are clogs the pathways making it difficult to remove the mucous (due to its abnormal consistency). Furthermore, a wide range of bacteria, fungi, and acari can stick to the mucus and cannot be eliminated. This results in reoccurring pneumonia and other bacterial infections, typically found in cystic fibrosis [21,23,36]. Additionally, Almeida et al. (2013) [91] analyzed the testicular tissue after biopsies from patients displaying abnormal spermatogenesis to describe the role of apoptosis in azoospermia. They conducted testicular treatment biopsies from 27 male patients. Five were cases with previously diagnosed oligozoospermia, nine with obstructive azoospermia (among them four patients with CBAVD), and in 13 cases non-obstructive azoospermia (5 men with hypo-spermatogenesis, there cases with sperm maturation arrest and five with Sertoli cell syndrome). These data focused on the activity of certain caspases: 8 and 9 which inaugurate the apoptotic pathways, as well as caspase 3, which determines the point of no return in apoptosis of cells. They found an increased activity of caspase 3 in Sertoli cell syndrome and germ cells with higher activity of caspases in hypo-spermatogenesis. In secondary obstructive disorders they noted diversified caspase activity, while in oligozoospermia significantly higher activity of caspase 9 in comparison to caspase 8 in spermatogonia was noticed. Finally, in primary obstructive disorders and hypo-spermatogenesis, caspases 3 and 9 showed significantly increased activity. That is why the importance of caspase-signalling pathways in human spermatogenesis is significant [91]. These authors point out that germ cells apoptosis is even necessary for normal spermatogenesis. The problems arise when the rate of sperm apoptosis is too high. The concentration of sperm decreases and abnormal seminal motility appears. Thus, these studies confirm a direct relationship between the apoptosis of germ cells and the failure of spermatogenesis.

### 4.3. Other Genetic Diseases Connected with Infertility: Klinefelter Syndrome and Kallmann Syndrome

Klinefelter syndrome and Kallmann syndrome are also considered common reasons for male infertility. Both diseases are connected with impairments of the X chromosome. The presence of an extra X chromosome in men, karyotype (XXY), is responsible for Klinefelter syndrome (47-XXY or XXY, i.e., the set of symptoms that occurs in two or more X chromosomes in males). The condition was first described in 1942. The symptoms include fibrosis of spermatic ducts, small testicles, azoospermia, and a decay of potency. In biochemical analysis Klinefelter syndrome patients display high levels of gonadotrophins and low levels of testosterone [28,36,92]. In Kallmann syndrome there are several possible mutated genes involved in pathogenesis. Mutations of the *KAL1* gene located on the X chromosome are most important. *KAL1* gene is located on the X chromosome at Xp22.3 and is affected in males with Kallmann syndrome. This gene codes for a protein of the extra-cellular matrix, anosmin-1, which is involved in the migration of nerve cell precursors (neuro-endocrine GnRH-cells). Deletion or mutation of this gene results in loss of the functional protein and affects the proper development of the olfactory nerves and olfactory bulbs. Neural cells that produce GnRH fail to migrate to the hypothalamus. However, other mutated genes are important, mainly fibroblast growth factor receptor 1 *FGFR1*, known as basic fibroblast growth factor receptor 1, fms-related tyrosine kinase-2/Pfeiffer syndrome, and CD331, as a receptor of tyrosine kinase, whose ligands are specific members of the fibroblast growth factor family. *FGFR1* has been shown to be associated with Pfeiffer syndrome. Moreover, the fibroblast growth factor 8 *FGF8* is a protein that is encoded by the *FGF8* gene, and protein coding gene *PROKR2* (prokineticin receptor 2) encodes a protein expressed in the supra-chiasmatic nucleus SCN circadian clock that may function as the output component of the circadian clock, and also WDR11 (WD repeat domain 11), known as bromodomain and WD repeat-containing protein 2 (BRWD2), a protein that is encoded by the *WDR11* gene. *WDR11* is a protein coding gene and PROKR2; a G protein-coupled receptor encoded by the *PROKR2* gene. Prokineticins are secreted proteins that can promote angiogenesis and induce smooth muscle contraction. These proteins encoded by *PROKR2* gene are membrane protein, which G protein-coupled receptor for prokineticins may contribute to manifestation of the condition. The symptoms of Kallmann syndrome include disorders of reproductive system (hypogonadism) with anosmia [32,34]. Thus while *PROK2* is type of gene mutation (protein coding gene; this gene encodes a protein expressed in the SCN circadian clock that may function as the output component of the circadian clock), *PROKR2* is a type of gene mutation (prokineticin receptor 2; a G protein-coupled receptor encoded by the PROKR2 gene in humans). The protein encoded by this gene is an integral membrane protein and G protein-coupled receptor for prokineticins.)

#### 4.3.1. Klinefelter Syndrome 

Høst et al. (2014) [30] defined Klinefelter syndrome as the most abundant sex-chromosome disorder, connected with hypogonadism and infertility. They state that this disease affects one in 600 men, but because of its high diversification in clinical presentation only 25% of men with Klinefelter syndrome are diagnosed with the disease. Among the typical symptoms of the condition they noted azoospermia, as well as various psychiatric problems (manifesting for instance in learning difficulties). However, the long term manifestations may encompass degradation in muscle mass and bone mineral mass, increased risk of diabetes type 2 and the threat of metabolic syndrome. In Klinefelter syndrome the loss of germ cells begins during the fetal period, continuing through infancy and intensifying in puberty. Fibrosis of the seminiferous tubules and a reduction in testis size are accompanied by long-lasting germ cell degradation [30]. Subsequently, the researchers described the appearance of adult patients with this syndrome as above average height, sparse body hair (due to androgen deficiency), narrow shoulders, broad hips, and small, firm testicles, while adding that deviations from that description are quite frequent. Nieschlag (2013) [29] remarked that the Klinefelter syndrome karyotype (47, XXY, aneuploidy of sex chromosomes) appears in up to 0.2% of male infants (one of the most frequent types of congenital chromosomal impairment). Among psychiatric aspects connected with the disease, they observed verbalization difficulties and problems with socialization among the youngsters. Furthermore, they described several pathological conditions accompanying Klinefelter syndrome including a lack of libido, erectile dysfunction, azoospermia, as well as gynecomastia, osteoporosis, thrombosis, and even epilepsy. Nieschlag (2013) [29] also mentioned that treatment of the disease is based on testosterone supplementation, instigated where low testosterone levels occur. He maintained that without proper treatment, as well as without treatment of the conditions accompanying Klinefelter syndrome (type 2 diabetes, varicose veins, embolism), the length of life of those patients may be up to 11 years shorter than the average age of male population. Simultaneously, Molnar et al. (2010) [26] stated that behavioral problems and learning delays in children often appear as the first step in this syndrome recognition. As proof the authors described the case of an 18 year old Somali boy with Klinefelter syndrome: recognition of the disease started with the observation of behavioral problems at school. During further investigation (determination of prolactine, testosterone, follicle-stimulating hormone, and luteinizing hormone levels, as well as the analysis of thyroid functionality and measurement of testis size) this syndrome was confirmed. Therefore, Molnar et al. (2010) [26] suggested that in cases of boys with learning problems, physicians should consider this syndrome as a possibility in their diagnosis. Some authors describe a range of treatment methods available for patients with Klinefelter syndrome who desire to have offspring. Certain amounts of testicular sperm can be retrieved surgically from the testis of adult men with this syndrome (testicular sperm extraction and intra-cytoplasmic sperm injection). There are also several techniques employed to increase testosterone levels, while classical testosterone supplementation supposedly even improves cognitive abilities in patients [26,30].

Gi Jo et al. (2013) [28] stated that Klinefelter syndrome is present in about 10% of azoospermic men. The frequency of morbidity amounts to 0.1–0.2% in general population whilst in 0.15–0.17% cases of the syndrome is recognized in prenatal diagnoses. The researchers tested over 18,000 pregnant women to detect Klinefelter syndrome in their offspring at the fetal stage. Twenty-two fetuses had Klinefelter syndrome, which was 0.12%, while after restriction of the group to only male features the proportional incidence was 0.23%. In the interpretation of their results Gi Jo et al. (2013) [28] note that fetal frequency of syndrome was higher than commonly observed. The researchers suspect that the possible reason for the occurrence of such a high syndrome level in features in their study was the advanced maternal age of mothers (over 35 years). They suggested that the risk of Klinefelter syndrome in offspring may increase with maternal age. Moreover, Turriff et al. (2011) [27] focused on psychiatric impairments accompanying this syndrome. They examined 310 participants of diverse age, from 14–75 years old. They analyzed the attitude of participants to such problems as perception of stigmatization, perceived negative consequences of karyotype XXY, and the matter of having children. Karyotype XXY is a Klinefelter syndrome known as 47, XXY or XXY, i.e., the set of symptoms that result from two or more X chromosomes in males. These authors established that nearly 70% of men with this syndrome displayed symptoms of depression and described several psychiatric manifestations associated with Klinefelter syndrome, including depression, anxiety, schizophrenia, psychoses, hallucinations, and paranoid delusions. They concluded that both adolescents and adults with this syndrome have an increased risk of psychiatric disorders. In their opinion, depression was the most important psychiatric symptom, appearing in syndrome, a condition which significantly decreases the quality of life of patients and may even lead to suicide [27]. Accardo et al. (2015) [92] considered the risk of testicular cancer in men with Klinefelter syndrome; adult patients with show testicular abnormalities such as fibrosis of the seminiferous tubules, hyperplasia of the interstitium, diffuse hyanilization, and cryptorchidism with a six times higher frequency than in the general male population. In addition to destructive changes in the testis, the authors describe several other diseases, possibly accompanying syndrome including venous disease, leg ulcers, and a higher morbidity due to certain malignant tumors, for instance malignancies in the lungs. These data analyzed the risk of testicular cancer in patients with Klinefelter syndrome. They measured several markers, such as serum levels of lactate dehydrogenase and alpha-fetoprotein. They conducted testicular ultrasound and in certain cases magnetic resonance imaging, and did not find increased signs of testicular cancer [92]. Accordingly, despite the risk of pathological conditions accompanying Klinefelter syndrome, the threat of testicular cancer appears to be low.

Additional disorders accompanying Klinefelter syndrome including abdominal obesity and metabolic syndrome were found by [93]. Eighty-nine adult patients had a higher risk of these conditions, but the researchers focused on younger patients, pre-pubertal boys, aged from 4–12.9 years old (measurements included height, weight, waist circumference, blood pressure, the concentrations of insulin, fasting glucose, and lipids). Compared to healthy controls, children with Klinefelter syndrome had wider waist circumference and engaged in less physical activity. Furthermore, in over one third of children, increased LDL cholesterol was noted, nearly one fourth had insulin resistance, and 7% fulfilled the criteria for metabolic syndrome diagnosis. Thus, Bardsley et al. (2011) [93] confirmed that certain disorders, which usually accompany this syndrome, may appear in youngsters. Additionally, Van Rijn et al. (2012) [94] examined the cognitive disorders which commonly appear in Klinefelter syndrome stating that the analysis of cognitive functionality of patients’ brains may deliver valuable information about neural mechanisms involved in social processing. In an experiment conducting a task based on judging facial expressions, men with this syndrome and healthy men were asked to assess faces as trustworthy or untrustworthy and asked to guess the age of the faces. During the first part of the task men obtained a lower valuation in several brain activities, including poorer screening of socio-emotional information (amygdala), poorer subjective emotional experience (insula), and poorer perceptual face processing (fusiform gyrus and superior temporal sulcus). During the second part of the task the perceptual face processing was also reduced in men with this syndrome. The studies elucidated direct relationships between abnormal social behaviors accompanying Klinefelter syndrome and a reduced functionality of the neural network [94,95,96].

#### 4.3.2. Kallmann Syndrome

Klinefelter syndrome, because of its relatively high frequency of occurrence in the human population, is well characterized. On the other hand, another genetically-determined condition, resulting in infertility, is Kallmann syndrome. This disease is caused by mutations of the KAL1 gene, located on the X chromosome. The symptoms appearing in men include small testicles, underdevelopment of the penis, delayed maturation, and a lack of a sense of smell. However, the maintenance of fertility in patients is possible [36,97,98]. Additionally, Quaynor et al. (2011) [33] stated that Kallmann syndrome is often connected with hypogonadotropic hypogonadism and anosmia. The fundamental impairments arise from low levels of sex steroids and low concentration of gonadotropins. In their opinion gonadotropin-realizing hormone GnRH appeared to be the most important hormone involved. It influences the hypothalamic-pituitary-gonadal axis functionality, playing an essential role in processes at puberty. When the secretion or the activity of GnRH is disturbed, pubertal disorders and reproductive impairments result. Both Laitinen et al. (2011) and Quaynor et al. (2011) [32,33] explained the reason for atrophy in the sense of small in the Kallmann syndrome. It is caused by cessation of GnRH neuronal migration within the meninges (GnRH, as well as olfactory neurons not reaching the hypothalamus). Furthermore, they expanded the list of possible manifestations of Kallmann syndrome to idiopathic hypogonadotropic hypogonadism. They added several impairments which were not connected with fertility, such as dental agenesis, midline facial defects, and even hearing loss. Laitinen et al. (2011) [32] admitted that an exact estimation of the incidence of Kallmann syndrome in human populations is difficult because the syndrome is clinically and genetically diversified. Nevertheless it seems to be 3–5 times more frequent in men than women. These researchers examined the Finnish population collating the phenotypic and genotypic features among patients with this syndrome, as well as the incidence of the disease in Finland. The frequency of Kallmann syndrome was different among men and women, being one case in 30,000 men versus one case in 125,000 women. They assessed the phenotypic reproductive features accompanying syndrome in a group of 25 men and five women. The phenotypes found were heterogeneous, ranging from partial puberty to severe hypogonadotropic hypogonadism. In an genetic analysis the authors focused on genes possibly contributing to this syndrome manifestation, i.e., *KAL1*, *FGFR1*, *FGF8*, *PROK2*, *PROKR2*, *CHD7* (chromodomain-helicase-DNA-binding protein 7, known as ATP-dependent helicase CHD7, is an enzyme that in humans is encoded by the *CHD7* gene). CHD7 is an ATP-dependent chromatin remodeler homologous to the *Drosophila* trithorax-group protein Kismet and WDR11, a type of gene mutation (WD repeat-containing protein 11, known as bromo-domain and WD repeat-containing protein 2 (BRWD2) is a protein that in humans is encoded by the *WDR11* gene). KAL1 mutation was detected in men, while FGFR1 mutation was noted in women and men. The results confirmed that it is difficult to give a clear diagnosis of Kallmann syndrome, because of the multitude of genetic factors contributing to the syndrome pathogenesis [32]. It goes far beyond these possible genes and is still waiting for further exploration.

On the other hand, Pedersen-White et al. (2008) [31] mentioned that the molecular basis for most cases of Kallmann syndrome and idiopathic hypogonadotropic hypogonadism is still unknown. Many mutations contributing to the disease remain undiagnosed. They suggested that the gonadotropin-releasing hormone receptor *GNRHR* gene (apart from *KAL1* and *FGFR1*) could also be related to Kallmann syndrome, but in their opinion mutations in the *GNRHR*, *KAL1*, and *FGFR1* genes account for only 15–20% of all possible reasons of idiopathic hypogonadotropic hypogonadism and Kallmann syndrome (*GNRHR* is a protein that is encoded by the *GNRHR* gene, which encodes the receptor for type 1 gonadotropin-releasing hormone). Pedersen-White et al. (2008) [31] conducted a screening study including 54 patients (men and women) with Kallmann syndrome and idiopathic hypogonadotropic hypogonadism. The results found that *KAL1* deletions appeared in 4 cases. After the restriction of the experimental group to anosmic men only, the result was four out of 33 patients. Thus, these researchers suggest that *KAL1* mutations are one of the most common reasons for Kallmann syndrome, but impairments in the other tested genes may also participate in the disease [31]. Similarly, Dodé and Rondard (2013) [34] remarked that the phenotype of Kallmann syndrome results from interruptions in the nerve fibers located in the nasal region, the olfactory, vomero-nasal, and terminal. The impact of these impairments is manifested as disturbances in the migration of gonadotropin-releasing hormone synthesizing cells between the nose and the brain. They discussed all genes connected with Kallmann syndrome that had been previously described, including *KAL1*, *FGFR1*, *PROKR2*, *PROK2*, *FGF8*, *CHD7*, *WDR11*, heparan sulfate 6-O-sulfotransferase 1 *HS6ST1*, and semaphorin-3A *SEMA3A* (a protein SEMA3A that in humans is encoded by the *SEMA3A* gene). HS6ST1 is the protein encoded by the gene *HS6ST1* and is a member of the heparan sulfate biosynthetic enzyme family. Heparan sulfate biosynthetic enzymes are key components in generating a myriad of distinct heparan sulfate fine structures that carry out multiple biological activities. This enzyme is a type II integral membrane protein and is responsible for 6-O-sulfation of heparan sulfate. This enzyme does not share significant sequence similarity with other known sulfotransferases). Dodé and Rondard (2013) [34] described the essential roles of these genes and assessed the proportion of Kallmann syndrome cases connected with their mutations. They found that *KAL1* contributes to an increase in the extra-cellular matrix glycoprotein anosmin-1, while *FGF8* and *FGFR1* encode fibroblast growth factor-8 and fibroblast growth factor receptor-1. *PROKR2* and *PROK2* are responsible for the generation of prokineticin receptor-2 and prokineticin-2. According to these authors’ assessment, mutations in *KAL1* appear in about 8% of cases of Kallmann syndrome, *FGF8* and *FGFR1* both appear in about 10% of cases and mutations both in *PROKR2* or *PROK2* are responsible for about 9% of cases. In addition, mutations in the *CHD7* gene lead to CHARGE syndrome (coloboma, heart defects, choanal atresia, retarded growth and development, genital abnormalities, and ear anomalies) in many patients accompanying Kallmann syndrome [34]. CHARGE syndrome, known as CHARGE association, is a rare syndrome caused by a genetic disorder. First described in 1979, the acronym CHARGE came into use for newborn children with the congenital features of coloboma of the eye, heart defects, atresia of the nasal choanae, retardation of growth and/or development, genital and/or urinary abnormalities, and ear abnormalities and deafness. These features are no longer used in making a diagnosis of CHARGE syndrome, but the name remains. About two thirds of cases are due to a *CHD7* mutation. Ultimately, practically all researchers agreed that, despite the estimated prevalence of this syndrome of one in 8000 men and nearly five times lower than this in women, the real frequency of the disease may be higher since so many of the genes potentially involved in Kallmann syndrome remain unexplored [31,32,33,34].

## 5. Summary and Conclusions

The data quoted in this review would agree that the pool of factors harmful to human health which has accumulated in the environment, is very large. Most of these factors affect the human reproductive system and fertility adversely [5,6]. Pb, Cd, Hg, Mo, and other heavy metals appear to be detrimental to sperm concentration and quality [1,52]. The authors expound a list of sperm and spermatogenesis depressors, describing the negative effects of dioxins, pesticides, phthalates, industrial solvents, as well as traffic fumes and food additives [4]. Obviously even house dust can modify reproductive hormone levels [3]. Researchers noted close relationships between many of the harmful substances mentioned above and increased oxidative stress. The problem of overproduction of ROS is usually connected with decreasing activity of certain antioxidative enzymes, such as catalase, superoxide dismutase, and glutathione peroxidase [7,10]. Many of these authors noticed certain behaviors that people can easily initiate on their own, such as a cessation of smoking or introducing a low-fat diet, can considerably reduce oxidative stress and improve reproductive condition [9]. A large pool of research has described the role of an anti-oxidative diet as an effective tactic in reducing oxidative stress. Beta-carotene, vitamin A, C, E, B complex, and lycopene have all been considered as beneficial factors in the lowering of oxidative stress markers and the improvement of anti-oxidative defense [12,13,15,16,78]. Another strategy aiding sperm quality appears to be supplementation of Zn and Se, which both improve semen concentration and motility [14].

Reactive forms of oxygen may cause destructive changes on a genetic level, for instance through DNA breakages and genetic factors were estimated to contribute to at least 5–10% of cases of male infertility [8,80]. We analyzed common genetic factors in male infertility, focusing on impairments in chromosomes Y, X, and 7. With respect to the Y chromosome, authors richly described the AZF region and microdeletions in domains AZFa, AZFb, AZFc, and AZFd [17,18,84]. It appears that a relatively minor manifestation of such deletions causes a lowering in the amount of sperm cells in semen, while the most serious deletions cause azoospermia [19,20,80]. The phenotypes vary between populations but micro-deletion and AZFc deletions are definitely the most frequent [86]. Male infertility also occurs in cystic fibrosis and the congenital bilateral absence of the vas deferens, both caused by mutations in the *CFTR* gene, located on chromosome 7. Obstruction of spermatic ducts by sticky mucus is a feature of cystic fibrosis, while aplasia of spermatic ducts applies to CBAVD. Regarding the common genetic cause of these conditions, CBAVD has been described as a form of expression of cystic fibrosis [22,23,25,36,89]. Finally, with respect to disorders associated with the X chromosome, Klinefelter syndrome, as one of the most frequent genetic causes of male infertility (1 in 600 men), is well characterized. The authors described genetic pathogenesis, the presence of an extra chromosome X in the male karyotype, as well as phenotypic manifestations, including small testis, azoospermia, degeneration of spermatic ducts, as sometimes coupled with psychiatric impairments and learning delays [26,27,28,29,30,93,94]. A well-characterized genetic disorder is Kallmann syndrome, where the condition results from mutations in various genes, including *KAL1*, *FGFR1*, or *FGF8*. It manifests as a combination of reproductive impairments (small testicles and delayed maturation) and the lack of a sense of smell [31,32,33,36]. The prevalence of this syndrome among male patients is estimated at 1 in 8000 but many genes possibly implicated in this disease are still unknown [34].

This review demonstrates that male health and fertility are directly connected with environmental conditions. We are exposed to various, potentially harmful, factors which intensify oxidative stress and decrease the natural defenses of the body. Subsequently, ROS damages the reproductive system and other essential systems and even causes impairments on a genetic level [8,97]. Further research should be undertaken to broaden our understanding of these environmental sources of immunogenetic disorders accompanying male infertility, in decreasing both lipoperoxidation and antioxidative activity. This will help determine the distribution and prevalence of potential risk factors in different regions. The results of future analysis should definitely improve the prevention of male infertility, as well as widen the diagnostic possibilities.

Summarizing: (1) Genetic factors are implicated in at least 10% of cases of male infertility [80]; (2) Amongst infertile men the frequency of *AZF* microdeletions is estimated at 7–8%, with a wide variation across populations [81,87]; (3) Alongside karyotype abnormalities (15% of azoospermic, 6% oligozoospermic cases), *AZF* microdeletions are considered as the second most common genetic reason of spermatogenic failure [18,20,83]; (4) Amongst various *AZF* genes the *DAZ* gene family is reported as the most frequently deleted *AZF* candidate [35]; (5) Screening of *AZF* microdeletions can be useful in explaining idiopathic cases of male infertility as well as in genetic consulting prior to assisted reproduction [87]; (6) An exact evaluation of how seriously pollutants and the destabilization of the elemental balance of the human organism lessen the quality of sperm and reduce male fertility should be conducted; (7) Studies of the induced oxidative stress and negative immunogenetic changes in the human reproductive system caused by toxic chemicals are important; (8) An evaluation of the significance of polymorphisms correlated with changes in reproductive potential and pro-anti-oxidative mechanisms as markers of pathophysiological disturbances of the male reproductive condition needs to be performed; (9) The inference from the relationships between environmental degradation and the occurrence of genetic diseases, connected with infertility, needs to be established.
